# Student Perceptions of the Implementation of Novel Sonography Phantoms in Radiography Curricula: A Mixed Methods Study

**DOI:** 10.1002/jmrs.70067

**Published:** 2026-02-03

**Authors:** Jad Boutros, Susan Said, Jillian Clarke

**Affiliations:** ^1^ Discipline of Medical Imaging Science, Susan Wakil Health Building The University of Sydney Camperdown New South Wales Australia

**Keywords:** education, imaging phantoms, radiography students, simulation, sonography

## Abstract

**Introduction:**

Sonography phantoms are an educational tool for training student sonographers and acquainting health science students to the conventions of sonography, which employs high frequency sound waves for soft tissue imaging. Phantoms are objects that mimic human anatomy and their appearance on sonography, enabling the practical application of theoretical skills in a simulated environment. As such, sonography phantoms are essential in providing an interactive experience for medical imaging students by consolidating theoretical knowledge and facilitating collaborative learning. This research study aims to analyse the experiences of diagnostic radiography students' use of four new sonography phantoms recently integrated into their curricula to examine the implementation of the phantoms in this environment which has not yet been researched.

**Methods:**

A mixed methods study was performed using an online Qualtrics survey composed of open and closed‐ended questions as well as focus groups.

**Results:**

Twenty‐three students completed the survey, of whom thirteen participated in focus groups. Most students indicated strong engagement with these learning tools, found them easy to use, and demonstrated an improved understanding of sonography. Key themes generated from the focus groups and open‐ended survey questions included active phantom engagement, appreciation of sonography, and its role in medical imaging and quality improvement.

**Conclusion:**

Students indicated a substantial level of enjoyment from phantom engagement and learning, describing a greater appreciation of sonography.

## Graphical abstract

Anthropomorphic sonography phantoms can assist radiography students with understanding the theoretical principles of sonography. We examined students' perceptions of participating in tutorials which required students to use four sonography phantoms. Students enjoyed utilising the phantoms and developed a greater appreciation of sonography from participating in the tutorials.

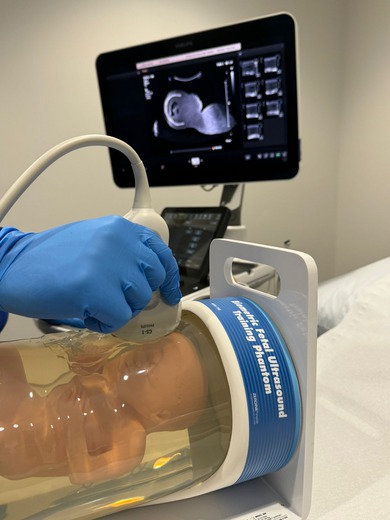

## 1 Introduction

Medical imaging is often the first point of care for patients who present with clinical symptoms with multiple differential diagnoses. Consequently, medical imaging staff require specialised training, starting at university, to set the foundation for the development of adaptable image acquisition skills in order to become competent, well‐rounded medical radiation science practitioners who can fulfil the growing demand for medical imaging in both private and public sectors [1].

The demand for medical sonography examinations, in particular, has increased by over 50% in Australia from 2012 to 2022, and in 2023 alone, over 12 million sonography procedures were processed by the Medicare Benefits Schedule [2, 3]. Sonography is listed on the Australian national shortage list [2, 3]. Alongside radiographers, sonographers perform a critical role in the diagnostic and treatment services offered in medical imaging departments. Radiographers work closely with sonographers when presented with dual sonography and radiography request forms. As such, radiographers must be able to understand the physical principles and uses of sonography as a part of their professional capabilities [4]. Beyond this, radiographers can improve the overall care they provide patients with a stronger understanding of sonography, whilst strengthenings their ability to collaborate with other clinicians.

Anthropomorphic phantoms are objects made from materials mimicking the properties of human tissue and replicate human anatomical structures. Sonography phantoms are used to simulate sonography examinations when educating medical imaging students [5]. Phantoms facilitate the refining of sonographic imaging skills and awareness of various aspects of patient anatomy [5].

While phantoms have evolved rapidly since their inception, they will continue to diversify into the future. The ideal teaching phantom is financially accessible, has tissue mimicking echogenicity, durability, and recyclability. The primitive blue phantom was an affordable gelatinous phantom which had a low background echogenicity and a hydrocolloid dressing which improved durability [6, 7]. Shalbi et al. used cadaver vasculature phantoms, employing human vessels, which attained near ideal sonographic attenuation characteristics which could be used for Doppler studies to enhance a user's ability to make blood flow measurements [8]. In contrast, dual phantoms, which can be used for both sonography and radiography imaging, provide the added benefit of a wider scope of educational effectiveness and more engagement through the use of multiple modalities [9].

Contemporary anthropomorphic phantoms are pedagogical tools which can simulate engaging and realistic learning opportunities for students [10]. The constructivist theory of pedagogy espouses the importance of students independently forming meaning for themselves, facilitated by making mistakes in a controlled environment [11]. Sonography phantoms offer the benefit of enabling students to make mistakes with no clinical ramifications, nurturing the successful, independent learning and critical thinking skills essential to a qualified practitioner [12].

A recent example in a simulation program by Mitchell et al. used a lower extremity venous phantom, arguing that the inability of the phantoms to dynamically demonstrate how students can avoid artefacts limited their educational effectiveness [13]. Hence, phantom simulation alone is not a substitute for clinical placement, which is essential to any radiography program [14].

**FIGURE 1:**
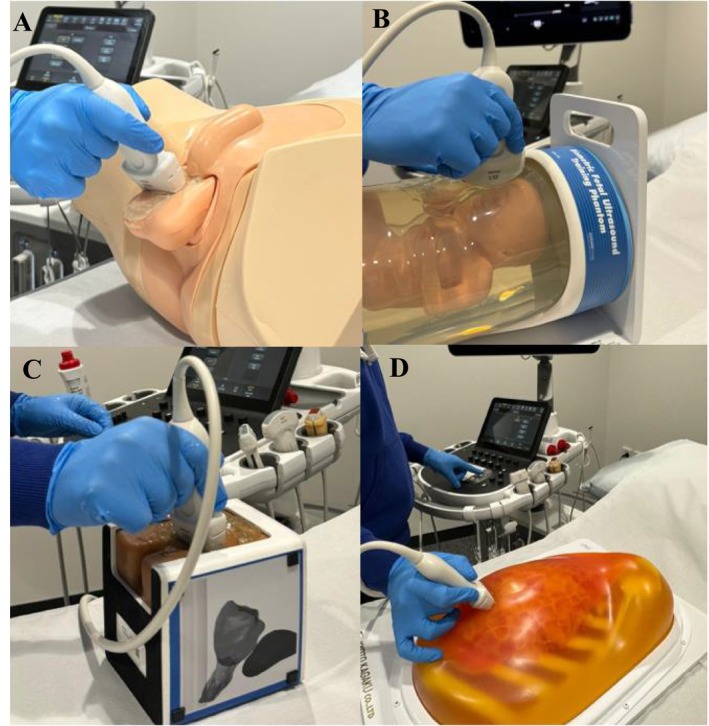
The anthropomorphic sonography phantoms implemented in tutorial classes for medical imaging students. (A) *Model Name*: Scrotal Ultrasound Phantom. *Model No*.: KKUS‐11. *Manufacturer*: Limbs & Things [15]. (B) *Model Name*: fetal ultrasound training phantom. *Model No*.: 068 Obstetric phantom. *Manufacturer*: Computerised Imaging Reference Systems [16]. (C) *Model Name*: Prostate Training Phantom. *Model No*.: 070L. *Manufacturer*: Computerised Imaging Reference Systems [17]. (D) *Model Name*: “BREAST FAN” Phantom. *Model No*.: US‐6. *Manufacturer*: Kyoto Kagaku [18].

**FIGURE 2:**
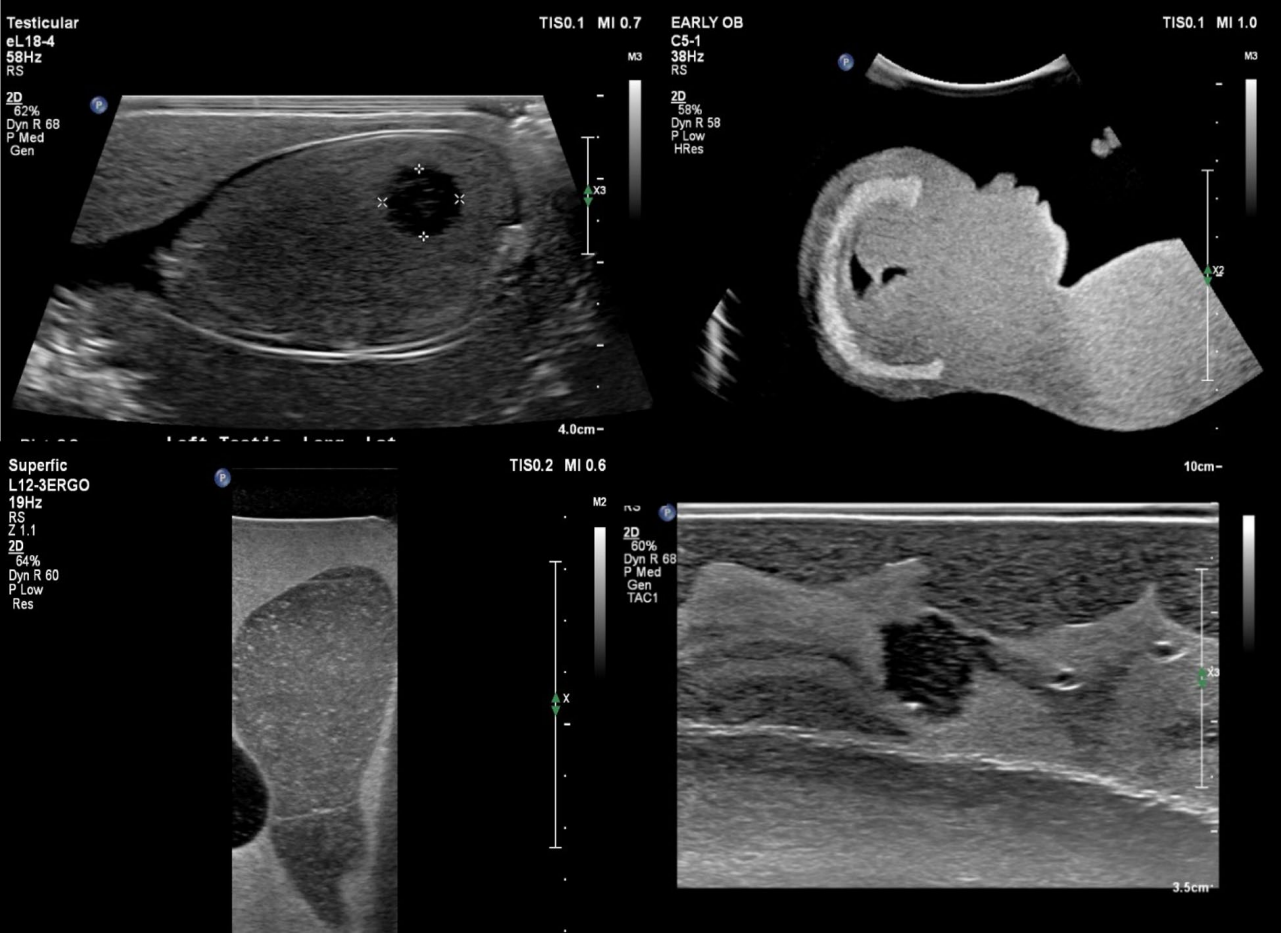
A scrotal (top left), obstetric (top right), prostate (bottom left) and breast (bottom right) sonogram, each generated from using their respective phantoms.

Recently, our Discipline purchased four new sonography phantoms, which demonstrate the anatomy of sensitive body parts, to acquaint medical imaging students with the background knowledge and uses of sonography, as a part of Domain 1(3) of their professional capabilities [4]. These anthropomorphic phantoms include obstetric/fetal, breast, scrotal and prostate models, which are used as an educational simulation tool to acquaint diagnostic radiography students with common sonographic applications. The four phantoms are demonstrated in Figure 1, and the sonograms which can be generated from each phantom are shown in Figure 2.

Because the phantoms shown in Figure 1 are novel, it is unknown whether they produce educational benefits for the student. Nor is the nature of the student's experiences known. Thus, this study explores the experiences of third‐year radiography undergraduate students' use of sonography phantoms by examining their potential educational benefits and any other potential benefits, such as developing in‐depth disciplinary knowledge and interprofessional collaboration.

## 2 Methods

### 2.1 Study Design

We conducted a mixed methods study. The first part of the study comprised a cross‐sectional survey questionnaire with demographic, Likert type, and open‐ended questions. The second part of the study comprised an adapted grounded theory analysis of qualitative focus group sessions. All of the data collected was original.

### 2.2 Population and Sample

Recruitment included any full‐time undergraduate third‐year student enrolled in the course who had used the sonography phantoms during a 2 h tutorial in one of their units of study. Undergraduate students could only use the sonography phantoms during this particular tutorial. Students were recruited as permitted by our ethics approval.

The population comprised 89 individuals. Non‐probability, convenience sampling was used to recruit students to maximise participation from this small population [19]. Twenty‐three participants completed the questionnaire, thirteen of whom attended a focus group.

### 2.3 Data Collection

Quantitative data was collected through a Qualtrics questionnaire. Likert‐type questions explored whether the students enjoyed using the phantoms and found them easy to use. The questions also examined students' perceptions of the usefulness of phantom simulation techniques in assisting a broader understanding of sonography.

The qualitative data was collected from both the Qualtrics questionnaire via descriptive open‐ended questions and through flexible questions asked of students by a facilitator in focus groups. An audio transcript was produced of each focus group using Zoom [20]. Since domineering students may undermine the ability of others to share their perspectives in a focus group, qualitative questions were also included within the survey to maximise the potential for data collection [21].

The Kirkpatrick model was employed as a framework for developing the qualitative focus group questions aiming to assess the usefulness of educational interventions [22]. The Kirkpatrick model is a training evaluation tool examining four key themes foundational to any learning experience: reaction, learning, behaviour, and results [22]. The main benefit of this model lies in its flexibility, which provides the interviewer with the ability to adapt the questions based on responses, making it an ideal framework for analysing diverse human learning experiences [23].

### 2.4 Setting

Students who completed the Qualtrics questionnaire could opt in to volunteer to participate in a focus group session at the end of the survey. Each of the three focus group sessions lasted between 15 and 30 min, and the participants in each group ranged from two to nine. The length of each session was dependent on the point at which data saturation was reached. Light refreshments and water were provided, and participants were reminded that they could withdraw at any time.

### 2.5 Statistical and Qualitative Analysis

The quantitative data collected from the questionnaire was analysed using descriptive and inferential statistics through the IBM SPSS Statistics software for Windows version 30 [24]. Firstly, data was screened for missing or implausible entries. No data estimates were made, as no data entries were implausible or incomplete and sensitivity analysis was not required. Median and mode of the responses of each question were calculated.

The qualitative data from both the Qualtrics questionnaire and focus groups were compiled and analysed through a three‐stage coding process performed by the first author using the Strauss and Corbin framework [25]. This framework is a detailed, systematic model with a high degree of analytical precision which outlines each of the three stages used: open, axial and selective coding [25]. It has been critically evaluated by researchers to be effective for grounded theory studies [26].

The first stage was open coding, where data was distributed into nominal categories based on the theme that each unit of data represents [25]. For instance, many of the students commented on how the phantoms were a practical and engaging way of learning about sonography. Thus, we grouped these quotes together under the open preliminary code ‘Engagement’.

In the axial coding stage, relationships were established between the codes to lay the foundation for the development of a summative explanation [25]. For example, we found that many participants who commented on the dynamic, real‐time nature of sonography added that this contributed to the engaging nature of the phantoms. A relationship was drawn between phantom engagement, enjoyment, and dynamic imaging, and these codes were grouped under the ‘Active Engagement’ theme.

In the final stage, the data was organised over a single, over‐arching theme which would take the form of an explanatory grounded theory [25]. However, this last stage was adapted, and a final summary was developed instead. As the sonography phantoms are novel and there is no pre‐existing data on their use as an educational tool for radiographers, we did not seek to develop a theoretical framework explaining student experiences [27].

The coding stages were flexible and not followed sequentially, meaning some of the themes from the preliminary open coding stage were recategorised based on the axial coding technique [28]. For instance, we initially categorised any student sentiments on real‐time imaging under the open code ‘Dynamic Imaging’. However, we discovered that many students made comparisons between radiography and sonography, which was a common axial code. As a result, we recategorised quotes referring to comparisons between the two under the open code ‘Connections with Radiography’, which was grouped under the axial code ‘Role of Sonography in Medical Imaging’.

### 2.6 Ethical Considerations

Students provided written consent after any questions they had were answered, and prior to focus group participation.

A single slide advertisement was used to recruit students to fill out the questionnaire and opt in to a focus group session which was placed around their classrooms. This was the only way students were recruited, meaning the authors, as students and teachers, could not participate or advertise recruitment themselves. Participants were asked not to disclose identifying information of participants known to have attended a focus group session.

## 3 Results

### 3.1 Participant Characteristics

Throughout April 2024, twenty students completed the Qualtrics questionnaire, of whom 30% participated in one of three on‐campus focus groups. One student was not included as they were an accredited sonographer, which could introduce a confounding variable into the data. Participant demographics are summarised in Table 1.

**TABLE 1:** Demographics.

	*n*	%	Median^a^	Mode
Age (range in years)				
18–24	21	92	X	X
25–34	1	4		
35+	1	4		
Gender				
Male	6	26		
Female	17	74		X
Experienced an ultrasound exam as a patient				
Yes	13	57		X
No	10	43		
Possessed sonography experience outside of placement				
Yes	2	9		
No	21	9		X
Time spent in sonography department on placement (days)				
0	12	52	X	X
1/2	10	44		
3–4 or more	1	4		

^a^ For nominal data (where responses cannot be ordered), the median cannot be found and was left blank.

### 3.2 Statistical Analysis

Overall, most participants either agreed or strongly agreed with each Likert statement. See Table 2.

**TABLE 2:** Participant ratings of phantom simulation.

	*n*	%	Median	Mode
Enjoyable				
Strongly disagree	0	0		
Disagree	0	0		
Neither agree nor disagree	2	9		
Agree	15	65	X	X
Strongly agree	6	26		
Learnt something new				
Strongly disagree	0	0		
Disagree	0	0		
Neither agree nor disagree	1	4		
Agree	6	26		
Strongly agree	16	70	X	X
Gained a broader understanding of sonography				
Strongly disagree	0	0		
Disagree	1	4		
Neither agree nor disagree	1	4		
Agree	7	31		
Strongly agree	14	61	X	X
Easy to use				
Strongly disagree	0	0		
Disagree	1	4		
Neither agree nor disagree	3	13		
Agree	12	52		
Strongly agree	7	31		

No significant correlations were found between the quantitative data.

### 3.3 Reflexivity

We acknowledge that the first author who conducted the focus group interviews was not independent of the data collected. As a final year radiography student, they were able to relate to many of the experiences of students, especially as they too had utilised the same phantoms for similar tutorials in the previous year. Their subjectivity, both as a radiography student and advocate for evidence‐based practice, pushed them to create an open, safe environment where participants felt free to share their perceptions. Additional follow‐up questions were asked on concepts described by participants requiring further clarification. Follow‐up questions were asked primarily when students presented positive or negative views of the phantom's design to better understand how the use of the phantoms could be improved. It was the familiarity of the interviewer with the subjects and subject matter that was foundational to creating trustworthiness as a qualitative researcher [29]. The researcher bias of the first author is also noted, as their own interest in the phantoms and their influence over the younger students could have made students speak more positively about the phantoms. To limit researcher bias, the second and third authors looked over each stage of coding for reliability checks before all three authors discussed each stage of coding in detail before data analysis was complete.

### 3.4 Themes

Focus group transcripts and open‐ended responses from the survey were first organised into 24 preliminary categories, pertaining to students' experiences, using NVivo [30]. This was the open coding stage. Four themes were generated based on relationships between the preliminary codes as a part of the second coding stage. Quotes labelled FG demonstrate comments from the focus group participants followed by their participant number.

#### 3.4.1 Theme 1: Active Engagement of Students in Learning

Across all three focus groups, and in the survey, participants referenced the active and engaging manner in which they learnt new skills from phantom use (*n* = 10), particularly those students who had spent a small amount of time on placement observing sonography examinations (*n* = 7). A representative response was given by one participant, who answered the survey question asking about any strong emotions they felt from using the phantoms, with:

I thought they were a fun way to interact … and learn about ultrasound.

#### 3.4.2 Dynamic Nature of Phantom Simulation

The practical nature of phantoms combined with real‐time sonography facilitated dynamic learning, where students could actively make connections between scanning techniques and image acquisition.

In X‐ray we can see in only a certain time. But in ultrasound, we can see the anatomy as it changes.

(FG1, P3)

#### 3.4.3 Engagement With Phantoms

Students felt that they were also a part of the learning experience. In essence, they had the independence to form an understanding of sonography procedures whilst being supervised by tutors:

This [obstetric] phantom is useful because we can see the same time and exact moment as we use the probe.

(FG3, P1)

The qualitative Qualtrics questions asked students about any learning they experienced from using the phantoms. One participant, with 3 days of prior experience in sonography while on placement, said they discovered how to:

Navigate in 3D space [inside each phantom] with [the ultrasound] transducer, and visualise the plane that the US beam cuts through. [I learnt] what anatomy looks like in US.

#### 3.4.4 Enjoyment of Sonography Tutorials

Students expressed sentiments of enjoyment and satisfaction from phantom use across the focus groups (*n* = 12). The open‐ended survey responses made substantial reference to the same sentiments (*n* = 11).

[The phantoms] were enjoyable just cause they were easy to use.

(FG3, P1)

In the survey, students were asked to describe any emotions they experienced from using the phantoms, to which a typical answer was:

I experienced curiosity and interest.

### 3.5 Theme 2: Confidence in Knowledge of Sonographic Appearances

Participants from the focus groups cited increased confidence in their ability to note the sonographic features of anatomy and pathology after using the phantoms (*n* = 6). Additionally, most survey participants referred to an enhanced appreciation of anatomical sonographic appearances (*n* = 18).

I can tell the difference between different anatomy on ultrasound after using the phantoms.

(FG1, P8)

#### 3.5.1 Anatomical Planes

Students highlighted a deeper understanding of how the structures they identified appeared on both transverse and longitudinal sonograms:

[I had a] better understanding of the different anatomical planes.

(FG2, P1)

#### 3.5.2 Anatomical Appearances

When prompted about any new learning experiences in the survey, students referred to a newfound appreciation of sonographic appearances:

[I understood] how to visualise the breast anatomy (ducts) and the orientation when scanning around the nipple.

[I understood] the shape, size and orientation of the testes when moving the probe around.

#### 3.5.3 Artefacts

Participants also cited an enhanced understanding of artefact formation which they learnt about from prior lectures. This was particularly the case for students who responded to the survey question asking participants to describe any new skills learnt:

Image artefacts actually exist in ultrasound.

Some participants wanted a greater emphasis on how to prevent artefacts when using phantoms:

I think it would have been cool if they told us how to find the artefacts and then negate that artefact.

(FG1, P7)

### 3.6 Theme 3: Role of Sonography in Medical Imaging

Some students referred to how sonography fits in broadly within medical imaging, as well as how it demonstrates diagnostic information that is supplementary to radiography (*n* = 5):

[I learnt how] to compare the appearance of different anatomical structures between radiographs and sonograms.

(FG1, P5)

A participant from the survey answered the question about new skills learnt, outlining they:

entirely understand the different orientations of ultrasound which is quite different to general X‐ray.

Furthermore, participants identified an increased confidence in the application of sonographic imaging:

I feel more confident in understanding how transducer types affect the images we get.

(FG2, P2)

I understand how certain pathologies and anatomy appear on ultrasound.

(FG1, P3)

### 3.7 Theme 4: Quality Improvement of Simulation Experience

Survey participants suggested different ways to improve the tutorials when prompted about extra materials that may be beneficial (*n* = 15):

Perhaps a labelling exercise through a website.

Maybe markers or labels that indicate the orientation more clearly (e.g., I found understanding the orientation of the obstetric phantom confusing).

In the focus groups, students also made suggestions for more demonstrations:

Perhaps a demonstration of the 12 o'clock sweep, like how to do the o'clocks would be have been better because what we found with our group was that it was very subjective.

(FG1, P1)

The supplementary materials were not enough.

(FG1, P3)

The supplementary materials provided included a labelling activity and detailed reference images for various sonographic projections acquired from each phantom. These materials asked students to obtain particular projections on each phantom, and to identify pathology.

### 3.8 Summary

Considering the diversity of themes surrounding student perceptions concerning phantom usage, a summary was drafted from the four key themes which replaced the selective coding stage of the Strauss and Corbin framework [25]. The summary is as follows:

We have found that the sonography phantoms are an engaging, educational tool which can increase confidence in radiography students' understanding of the role of sonography in medical imaging. We also found sonography phantom simulation can be improved with more supplementary guidance materials and an opportunity for familiarisation with clinical sonography before the tutorials.

**FIGURE 3:**
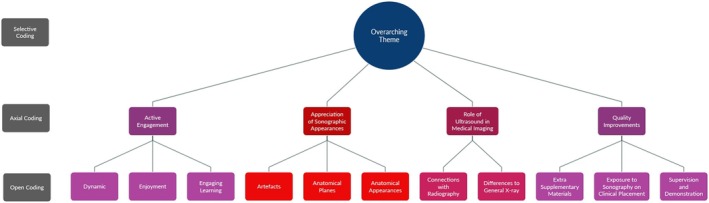
Key themes extracted through qualitative coding stages.

The connections drawn between each stage of thematic coding are demonstrated in Figure 3.

## 4 Discussion

Phantoms are a popular simulation tool for medical imaging students. Our study demonstrates that phantoms can increase confidence and preparedness for professional practice. This was demonstrated in a randomised trial by Mitchell et al. who measured higher scanning assessments of sonography students who practiced with a phantom compared to students who had no simulation preparation [13]. It is also noted that our study generated similar results in that some participants could not demonstrate artefacts from these phantoms, which can limit the overall usefulness of the phantoms [13]. Nonetheless, the study supports our conclusion that the potential for acquiring new knowledge from phantoms is valid [13].

In particular, sonography phantoms have been promoted as an essential form of radiology simulation given the growing demand for sonography training [2, 31]. Our novel study provides a guide to student perceptions of the integration of sonography simulation into radiography curricula. The survey noted that not all students found the phantoms easy to use, particularly students with no prior experience with sonography. As quality improvement was a key theme, it was clear that supplementary materials, such as labelling exercises, would help increase their ability to engage with the phantoms during tutorials. Chau et al. echo the importance of supplementary materials, arguing that the preparation of students is a critical aspect of effective simulation [32]. However, it is essential to consider the associated costs [33]. An academic confident with using the sonography phantoms could prepare an introductory lecture to prepare the students before their tutorials. However, this requires extra time to prepare, and only one academic in the department is capable of producing such a resource. Furthermore, providing an online interactive labelling activity designed to match the phantoms as a resource for revision after the tutorials can be challenging. This requires extra resource allocation and substantial amounts of time and effort to prepare.

Similarly, a study detailing the requirements of sonography simulation underscored the importance of phantom technology as a supplement to clinical placement, not a replacement [34]. Whilst confined to medical students, it demonstrated a substantial level of enjoyment and satisfaction by users, reflected by 82% of our survey participants who agreed that the phantoms were an engaging pedagogical tool. Furthermore, enjoyment of phantom use was a common code generated from the qualitative analysis.

During clinical placement, it may be difficult to allow students to practice their skills when sites are busy and have minimal supervision, or if staff are unwilling to mentor students. During their tutorials, students had the opportunity to take enough time to appreciate the sonographic appearances of the four phantoms and identify simulated pathologies that may be difficult to observe at placement sites. Consequently, students can have deeper learning and more time to reflect on the uses of sonography. Reflection is an essential stage of the Kirkpatrick learning theory [22]. This was seen most clearly amongst students who outlined their new learning and how their perceptions of sonography have changed as a result.

Gibbs, however, underscored the importance of sustained clinical placement blocks playing the greatest role in enabling practitioners to meet their professional capabilities, despite the benefits of increased confidence and reduced stress from simulation [35]. The phantoms aim to assist radiography students to build a sufficient foundation in the principles of sonography in line with their curriculum, which does not require sustained clinical placement blocks. Clinical experience would be essential for sonography students who have a different curriculum to radiography students.

Whilst radiography can work in symbiosis with sonography, each modality is unique and is used for differing purposes. Radiography students with little exposure to sonography can often find sonograms unfamiliar. These students may find sonography challenging to navigate or understand. Thus, student engagement with the phantoms is substantially influenced by their prior knowledge of the anatomical regions the phantoms represent, as well as their sonographic appearances. This is confirmed by cognitive learning researchers Brod et al., who argue that accumulated knowledge influences future learning experiences [36].

### 4.1 Limitations

There are several factors that limit the validity of this study. For the survey, the sample size was small due to the limited numbers of third‐year radiography students. Moreover, participants were recruited through convenience sampling, which introduces selection bias. Consequently, participants with positive experiences from the phantoms were more likely to opt into the study, which limits its generalisability significantly.

We implemented qualitative focus groups to holistically capture student perceptions of sonography phantoms in an effort to support, explain and provide rich data to add to the statistical findings from the survey, which lacked generalisability. The interviewer had to ensure that participants had no new insights to share in order to achieve saturation. This was particularly difficult for two of the focus groups, which had a limited number of participants.

### 4.2 Future Research

Our study demonstrated that sonography phantoms have shown the potential to provide educational benefit and professional development for third‐year radiography students. Further research could include greater sample sizes from multiple universities from both undergraduate and postgraduate radiography courses. It may also be worthwhile for students to observe sonographic exams prior to using the phantoms. To foster interprofessional collaboration, research into dual phantom simulation where sonography and radiography students work together to diagnose patients in a simulated hospital scenario would be interesting. Students could be assessed on the care they provide based on how well the images they acquire demonstrate the pathology, and could be asked to attend a focus group to share their experiences. This could demonstrate whether phantoms can allow students to practically apply their knowledge of other modalities. Further, research into the economic viability of phantoms could assist academic staff to plan a larger scale integration of sonography phantoms into radiography curricula, given our research demonstrated unique benefits for radiography students learning with the sonography phantoms.

## 5 Conclusion

This research provides a novel insight into students' perceptions of sonography phantoms within radiography curricula. Phantoms can be an enjoyable, engaging, and useful tool for teaching radiography students about the uses of sonography. Sonography phantoms can demonstrate the dynamic nature of sonography and how it complements radiography. When designed with adequate supplementary materials, phantoms can be a practical teaching tool to assist students in understanding the theoretical principles of sonography.

## Author Contributions

Study conception and design: Jad Boutros, Susan Said and Jillian Clarke. Data analysis: Jad Boutros, assisted by Susan Said and Jillian Clarke. Article writing, editing, final critical review for important intellectual content and approval of the final version: Jad Boutros, Susan Said and Jillian Clarke.

## Funding

The authors have nothing to report.

## Disclosure

No use of any form of artificial intelligence (including translation, paraphrasing or editing software or generative AI programs) was used in this research or the production of this manuscript.

## Ethics Statement

Ethical approval was obtained from The University of Sydney, Project no. 2024/HE000021.

## Conflicts of Interest

The authors declare no conflicts of interest.

## Data Availability

The data that support the findings of this study are available on request from the corresponding author. The data are not publicly available due to privacy or ethical restrictions.
